# Why patients with *THBD* c.1611C>A (p.Cys537X) nonsense mutation have high levels of soluble thrombomodulin?

**DOI:** 10.1371/journal.pone.0188213

**Published:** 2017-11-16

**Authors:** Yohann Jourdy, Nathalie Enjolras, Sandra Le Quellec, Jean Claude Bordet, Claude Négrier, Christine Vinciguerra, Yesim Dargaud

**Affiliations:** 1 Hospices Civils de Lyon, Centre de Biologie et Pathologies Est, Service d’hématologie Biologique, Bron, France; 2 EAM 4609 Hémostase et cancer, Université Claude Bernard, France; 3 Hospices Civils de Lyon, Hôpital Cardiologique Louis Pradel, Unité d'Hémostase Clinique, Bron, France; King Abdullah International Medical Research Center, SAUDI ARABIA

## Abstract

**Background:**

Recently our group has described a new autosomal dominant bleeding disorder characterized by very high plasma levels of soluble thrombomodulin (TM). The *THBD* c.1611C>A (p.Cys537X) mutation in heterozygous state was found in the propositus. This mutation leads to the synthesis of a truncated TM which has lost the last three amino-acids of the transmembrane domain and the cytoplasmic tail.

**Objective:**

We investigated the mechanism responsible for TM shedding in endothelial cells with *THBD* c.1611C>A mutation.

**Methods:**

Complementary DNA of TM wild type (TM-WT) was incorporated into a pcDNA3.1 vector for transient transfection in COS-1 cells. Mutagenesis was performed to create the c.1611C<A (TM_1-536_) mutant and 4 other TM mutants (TM_1-515_, TM_1-525_, TM_1-533_ and TM_1-537_) with a transmembrane domain having different lengths. The effect of shear stress, metalloprotease inhibitor, certain proteases and reducing agents were tested on TM shedding.

**Results:**

Western blot and immunofluorescent analysis showed that TM_1-536_ was produced and a certain amount of TM_1-536_ was anchored on the cell membrane. A significantly higher levels of soluble TM was observed in the TM_1-536_ cell medium in comparison with TM-WT (56.3 +/- 5.2 vs 8.8 +/- 1.6 ng/mL, respectively, *p* = 0.001). The shedding of TM_1-536_ was 75% decreased in cells cultured in the presence of a metalloprotease inhibitor. No difference was observed between TM_1-536_ and TM-WT shedding after cell exposure to cathepsin G, elastase, several reducing agents and high shear stress (5000 s^-1^). Significantly higher levels of soluble TM were observed in the cell media of TM_1-533_, TM_1-525_, TM_1-515_ in comparison with TM-WT (*p* < 0.05).

**Conclusion:**

The mechanism responsible for TM shedding is complex and is not completely understood: higher sensitivity of the TM_1-536_ to the proteolysis by metalloproteases and a defect of synthesis due to the decreased size of the transmembrane domain might explain the high levels of soluble TM in plasma of the carriers.

## Introduction

Thrombomodulin (TM, CD 1441) is a 557 amino acid type-1 transmembrane glycoprotein expressed at high levels on vascular endothelial cells [1]. This protein is encoded by an intronless gene (*THBD*) located on the chromosome 20p12-cen [2]. The mature protein have a molecular weight ranging from 70 to 100 kDa [3] and has an amino terminal lectin-like domain, a chain of 6 epidermal growth factor-like domains, a serine threonine rich region containing chondroitin sulfate glycosaminoglycan, a transmembrane domain and a short cytoplasmic tail [1,4]. The anticoagulant function of TM is mediated by its interaction with thrombin and protein C (PC). Thrombin-TM complex activates PC and thrombin-activatable fibrinolysis inhibitor (TAFI) [4,5]. Activated PC formation prevents the amplification of thrombin generation via proteolysis of activated coagulation factors V and VIII and TAFI down-regulates fibrinolysis. In addition to anticoagulant properties, TM exhibits other functions such as anti-inflammatory properties and role in embryogenesis [1].

TM also exits in a soluble form in plasma and urine, but in healthy human subjects, the circulating levels of soluble TM (sTM) are low (<10 ng/mL) [6]. Soluble TM is generated by enzymatic or chemical cleavage of the membrane bound-protein [7–9]. Several pathological conditions are associated with moderate increase of TM levels in plasma which has been suggested to be a marker of endothelial damage [10,11].

Recently, our group has described a family with an autosomal dominant disorder characterized by trauma- and surgery-related severe bleeding despite normal preoperative hemostasis tests. This bleeding disorder is characterized by very high levels of sTM in patient’s plasma e.g. 180 times higher than normal controls [12,13]. Using whole exome sequencing, the underlying genetic defect has been identified as the *THBD* c.1611C>A mutation (p.Cys537Stop), which is responsible for the synthesis of a truncated TM which has lost the last three amino-acids of the transmembrane domain and the cytoplasmic tail. The aim of the present study was to investigate the mechanisms responsible for excessive TM shedding from endothelial cells of patients with *THBD* c.1611C>A nonsense mutation.

## Materials and method

### Plasmids for *in vitro* expression

Full-length TM cDNA (TM-WT) was cloned in pCDNA3.1 plasmid vector (Invitrogen, Carlsbad, CA, USA). Several TM mutant plasmids, with nonsense mutation, were generated through site-directed mutagenesis using the QuickChange XL reagent (Stratagene, La Jolla, CA, USA) according to the manufacturer’s instructions. The substitutions were confirmed by DNA sequencing. The TM_1-536_ mutant corresponds to the proband’s mutation c.1611C>A (p.Cys537X). The TM_1-515_, TM_1-525_, TM_1-533_ and TM_1-537_ mutants correspond to truncated TM molecules with a transmembrane domain having variable lengths.

### Cell expression

COS-1 cell line (ATCC, CRL-1650) were grown at 37°C with 5% CO2 in Iscove’s essential medium (IMDM, GIBCO BRL, Gaithersburg, MD, USA) containing 25mM HEPES, 2mM L-Glutamine, 10% fetal calf serum (FCS) and 1% Penicillin-Streptomycin. Transient transfection were performed in 6 or 24 well plates (5.2X10^4^ cells / cm^2^) using X-tremeGENE9 DNA (Roche Molecular Diagnostic, Meylan, France) pre-incubated with 0.2μg/10^5^ cells of plasmid vectors, in serum-free IMDM. Analysis of transient expression was performed 36 to 48 hours after expression.

### Western blot analysis

Thirty-six hours after transfection, the medium was removed, changed by serum-free IMDM. One night latter, the medium was recovered, aliquoted and stored at -20°C. For cell lysis, cells were washed twice in phosphate-buffered saline (PBS, GIBCO BRL) and lysis buffer (HEPES pH 7.5 50mM, KCl 0.1M, MgCl2 2mM, Triton X-100 0.5%) containing protease inhibitors (Complete Mini, Roche, Bâle, Suisse) was added to each plates and incubated on ice 20 minutes (min). Then, the lysate was clarified with a centrifugation step and stored at -20°C.

For western blot analysis, conditioned medium was concentrated using Amicon Ultra 0.5mL centrifugal filter 10K (Merck KGaA, Darmstadt, Deutschland). After a SDS-PAGE step, western blotting was used to visualize TM molecules in cell lysate and conditioned medium. Thrombomodulin was immunodetected using mouse monoclonal anti human TM antibody (anti-thrombomodulin antibody [PBS-02] ab7640, Abcam, Cambridge, UK) and Horse Radish Peroxidase (HRP)-conjugated polyclonal anti-mouse antibody (Goat Anti-Mouse IgG (H+L)-HRP Conjugate, Biorad, Hercules, CA, USA). The kit Immun-Star™ (Western C™ Chemiluminescent, BioRad) was used to reveal TM.

### TM enzyme-linked immunosorbent assay (ELISA)

Media and cell lysate TM levels were quantified using the Thrombomodulin human ELISA Kit according the manufacturer’s instruction (Abcam).

### Enzymes and reducing agents for TM shedding experiments

Reduced glutathione (GSH), N-acetyl-L-cysteine (NAC), human leucocyte elastase and Cathepsin G from human neutrophils were purchased from Sigma-Aldrich (Saint-Louis, MI, US). GM6001 was from Calbiochem (San Diego, CA, US).

### Shear stress experiments

For experiments evaluating the effect of shear stress, 1.25.10^5^ COS-1 cells were cultured on 24-well plate and transiently transfected as previously described. Forty eight hours after transfection, shear stress (5000s-1) mimicking conditions in capillaries was applied to the cells during 0, 2, 5 or 10 min using cone and plate viscometer. Then, the concentration of TM was measured in supernatant.

### Immunofluorescent labelling and confocal microscopy analysis

The cells were cultured on Labtek chambers (Nalge Nunc, Naperville, USA). The cells were washed three times in PBS and fixed for 15 min in PBS containing 4% paraformaldehyde at room temperature. After three PBS washes, the cells were blocked with IMDM containing 0.5% of bovine serum albumin (BSA) for 30 min. Mouse monoclonal antibodies against TM (Abcam) were diluted 1:1000 in IMDM containing 0.5% BSA and incubated for 1h at room temperature. After 3 PBS washes, cell membranes were labeled with Alexa Fluor 594^®^ conjugated WGA (Wheat Germ Agglutinin conjugate, Invitrogen) diluted 5μg/mL in IMDM containing 0.5% BSA and incubated for 10 min at room temperature. Then, the chambers were washed 3 times with PBS before incubation for 30 min at room temperature with the secondary antibody Alexa Fluor 588^®^ conjugated anti-mouse immunoglobulin (Invitrogen) diluted 1:800 in IMDM containing 0.5% BSA. The slides were washed 3 times in PBS before being mounted in Dako fluorescent mounting medium and images were analyzed using a Leica SP5 X microscope (Leica, Wetzlar, Germany).

### Thrombin generation assay

The inhibitory effect of sTM on the coagulation capacity of normal plasma from controls was evaluated by Thrombin generation assay (TGA). Thrombin generation was measured using the Calibrated Automated Thrombography method (CAT, Stago, Asnières, France) using a low concentration of tissue factor 1pM (Innovin, Siemens, Marburg, Germany) and phospholipids vesicles 4μM (Avanti Polar Lipids, Alabaster, AL, USA) following the protocol previously described [14,15]. The main parameter of TGA used in this study was the Endogenous Thrombin Potential (ETP). ETP is the area under the thrombin generation curve. It has been previously shown that ETP is better correlated to the clinical outcome of patients with coagulation disorders compared to routine coagulation tests [16,17].

### Statistical analysis

The statistical analysis was performed using GraphPad Prism Instat 3.0 software (La Jolla, CA, USA). Means (m), variances and standard error (SEM) were calculated for data sets and all results were expressed as mean +/- SEM. Comparison between mean values was performed with a Student t-test. A *p value* < 0.05 was considered statistically significant.

## Results

### Expression of TM_1-536_ (p.Cys536X) in COS-1 cells

**Fig 1:**
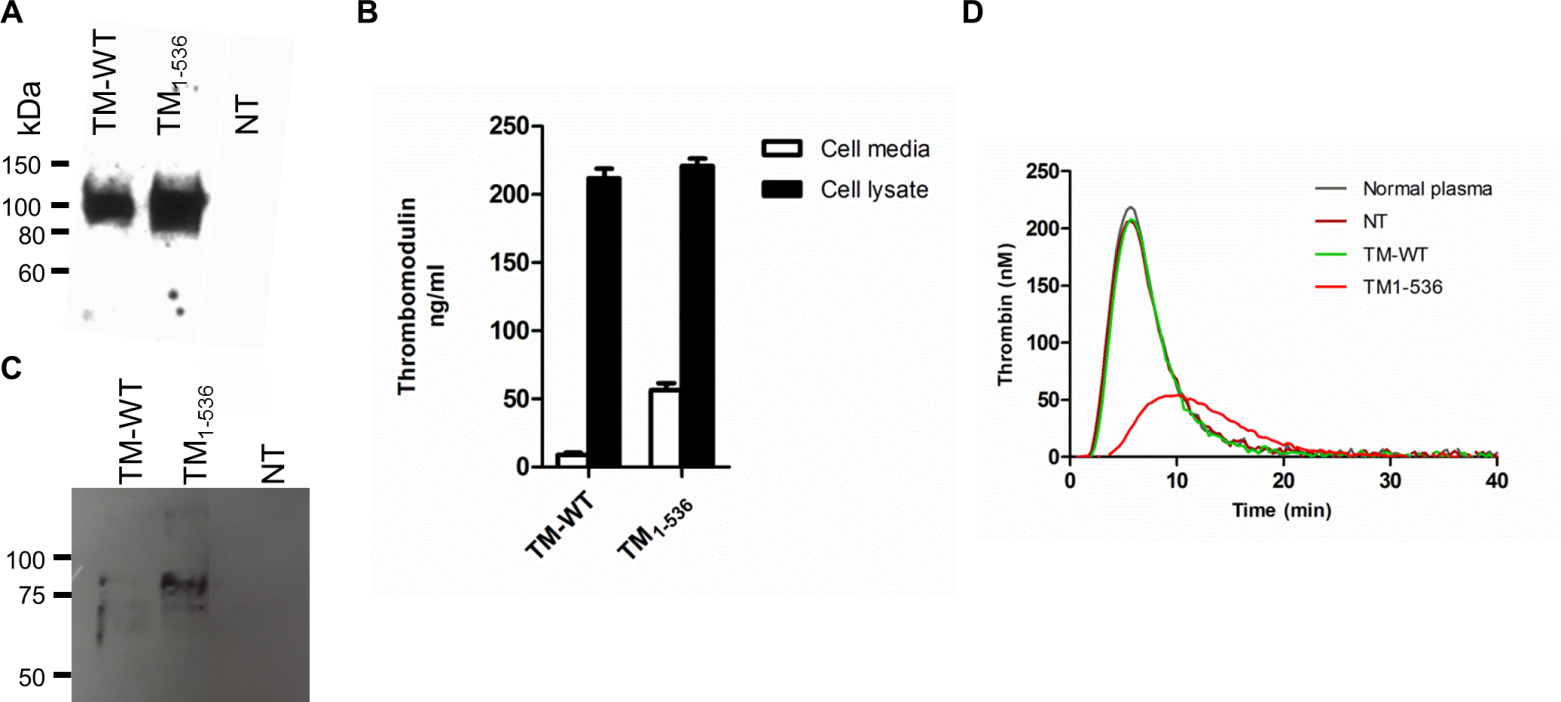
Characterization of the secreted and intracellular thrombomodulin from COS-1 cells. (A) Cell lysates western blotting of the expressed thrombomodulin constructs. SDS-PAGE under reducing conditions and immunoblotting of cell lysates expressing each of the mutant p.Cys536X (TM_1-536_), the wild-type thrombomodulin (TM-WT) or the non-transfected cells (NT). Immunodetection was performed with mouse monoclonal anti-TM antibody followed by HRP-conjugated anti-mouse antibody and enhanced chimoluminescence detection. (B) Thrombomodulin ELISA dosages in cell lysates and supernatants. (C) Cell supernatants western blotting (reducing conditions) of the expressed thrombomodulin constructs. (D) Thrombomodulin activities in cell supernatant. Thrombomodulin activities were evaluated measuring the decrease of thrombin generation of normal platelet poor plasma.

The mutation c.1611C>A (p.Cys537X) in the TM gene is responsible for a truncated TM molecule that has lost the last three amino-acids of transmembrane domain and the cytoplasmic tail. TM-WT and TM with c.1611C>A mutation (TM_1-536_) were transiently expressed in COS-1 cells. Forty eight hours after transfection, western blotting of cell lysates and supernatants were performed using a monoclonal anti-TM antibody. A band of 100 kDa corresponding to TM was detected in the cell lysate for both TM-WT and TM_1-536_ cells (Fig 1A). The TM antigen levels in the cell lysate measured by ELISA showed similar levels for each TM constructs (211+/-7 vs 220 +/-5 ng/mL for TM-WT and TM_1-536_ respectively, *p* = 0.37) (Fig 1B). However, a significantly higher levels of sTM was observed in the TM_1-536_ cell media in comparison with TM-WT (56.3+/-5.2 vs 8.8+/-1.6 ng/mL, respectively, *p* = 0.001) (Fig 1B). These results were confirmed by western blot analysis of the TM present in concentrated cell media (Fig 1C). A single band between 75-100kDa corresponding to TM_1-536_ was observed. No band was observed for TM-WT. These results demonstrated that TM_1-536_ was correctly synthesized by transfected COS-1 cells and partly secreted in the cell supernatant.

**Fig 2:**
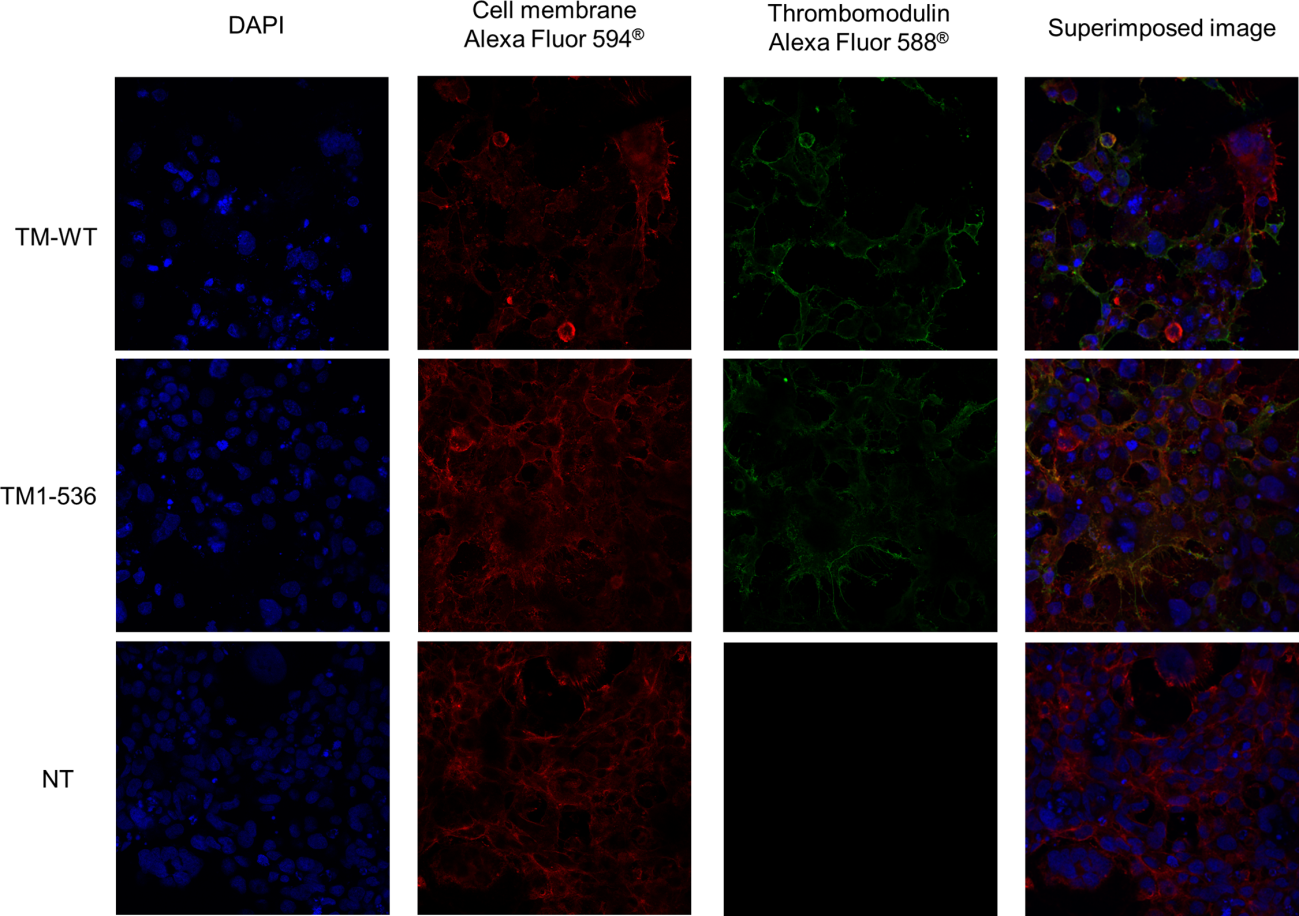
cell surface thrombomodulin immunofluorescent labelling and confocal microscopy analysis of transfected COS-1 cells. COS-1 cells were transiently transfected by TM-WT and TM_1-536_ expression vectors and were incubated in Labtek chambers for 48h. The immunofluorescent labeling was performed as described in the materials and methods section. Cell nuclei were labeled using DAPI (blue). Cell membranes were labelled using Alexa Fluor 594® conjugated Wheat Germ Agglutinin (red) and thrombomodulin were labelled using Alexa Fluor Fluor 588® antibodies (green). Superimposed images were obtained and revealed that both TM-WT and TM_1-536_ were expressed at the cell surface of COS-1 cells.

Cell surface expression of TM-WT and TM_1-536_ was studied using immunofluorescent labeling and confocal microscopy analysis. Representative several immunofluorescence images with labelling for TM (Alexa Fluor 594^®^) and cell membrane (Alexa Fluor 588^®^) are presented in Fig 2. For both TM-WT and TM_1-536_ cells, superimposition of Alexa Fluor 594 labelled cell membranes and Alexa Fluor 588 labelled TM revealed yellow area demonstrating the expression of TM on the cell surface. This result confirmed that some TM_1-536_ was anchored on the cell membrane whilst a certain amount of the protein was secreted in the supernatant.

### Secreted TM_1-536_ conserved anticoagulant activity

Secreted TM activity was evaluated by measuring the decrease of thrombin generation of a normal control plasma (ETP = 1291 nM.min) by adding the same volume of supernatants from TM-WT and TM_1-536_ cell cultures (Fig 1D). No significant decrease of thrombin generation was observed when 10 μL of TM-WT or non-transfected cell media were added to 80μL of normal PPP (ETP = 1269 and 1204 nM.min, respectively). A decrease of 54% was observed when TM_1-536_ cell supernatant was added to normal PPP (ETP = 595 nM.min). Thus, we showed that TM_1-536_ released into the cell supernatant was enzymatically active.

### TM_1-536_ does not exhibit a higher sensitivity to the proteases cleavage and reducing agents

**Fig 3:**
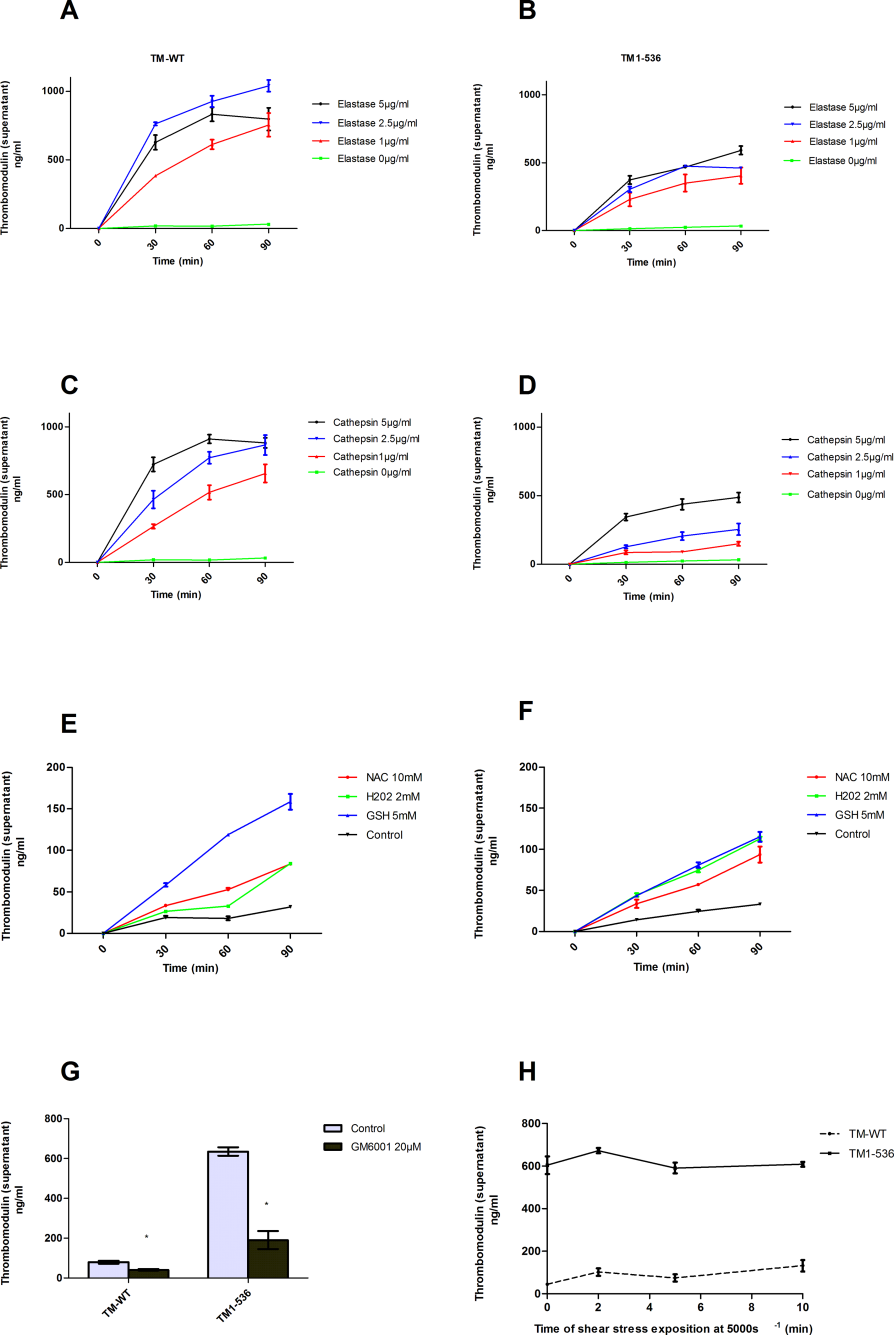
Thrombomodulin shedding under different conditions. Levels of released of thrombomodulin into cell culture medium were measure by ELISA. (A to D) Effect of proteases on TM-WT and TM_1-536_ release. Cells were treated with elastase and cathepsin G at final concentration of 1, 2.5 and 5 μg/mL for 90 minutes. (E and F) Effect of reducing agents on TM-WT and TM_1-536_ release. Cells were treated with 2mM H_2_O_2_; 10mM N-acetyl-L-cystein (NAC) and 5mM reduced glutathione (GSH) for 90 minutes. (G) Effect of GM6001, a broad-spectrum metalloproteinase inhibitor, on TM-WT and TM_1-536_ release. COS-1 cells were cultured during 48h after transfection in medium containing GM6001 (20μM). Results are presented as means +/- standard error (n>3). * p<0.05 versus control without protease inhibitor. (H) Thrombomodulin shedding after shear stress exposition. Forty-eight hours after transfection by TM-WT and TM_1-536_ expression vectors, COS-1 cells were exposed to shear stress (5000s^-1^) using “cone and plate” viscometer during 0, 2, 5 and 10 minutes. Thrombomodulin concentrations in the cell supernatants were measured after exposure to shear stress in order to evaluate the thrombomodulin shedding.

A low concentration of sTM is physiologically present in plasma. It’s generated by enzymatic cleavage and by the action of reducing agents. We hypothesized that the TM_1-536_ having a shorter transmembrane domain, might have a higher sensitivity to enzymatic or chemical cleavage compared to TM-WT. Forty-four hours after transfection, the medium was removed and replaced by FCS free medium containing different concentrations of proteases (cathepsin G and elastase at 1, 2.5 and 5 μg/mL) or reducing agents (NAC 10mM, H_2_O_2_ 2mM and GSH 5 mM). TM release in cell supernatant was measured at 30, 60 and 90 minutes (min) after incubation at 37°C. TM-WT and TM_1-536_ were very rapidly shedded in the supernatant after incubation with elastase and Cathepsin G. A significant release of membrane TM was detected 30 min after exposure to the enzymes (Fig 3A, 3B, 3C and 3D). The majority of TM was retrieved in the first hour of incubation. The concentration of TM_1-536_ in the supernatants was lower than the TM-WT concentration because a part of TM_1-536_ was spontaneously released into the media before the addition of the enzymes and eliminated when the medium has been replaced by serum-free medium. Exposure of COS-1 cells to the reducing agents such as NAC, GSH and H_2_O_2_, during 90 min, increased the shedding of both TM-WT and TM_1-536_ (Fig 3E and 3F). Very low concentrations of sTM were found in the control culture medium related to physiological cell turnover.

### Metalloprotease inhibitor importantly decreased the TM_1-536_ shedding

Several metalloproteases have been reported to be involved in ectodomain shedding of transmembrane proteins. In order to evaluate the potential role of metalloproteases in the TM_1-536_ shedding, cells were cultured in medium containing GM6001 (20μM), a broad spectrum metalloprotease inhibitor (against a disintegrim and metalloproteases, ADAMs, and matrix metalloproteases, MMPs). Forty eight hours after transfection, TM release in cell supernatant was measured. A decreased of 75% of the shedding of TM_1-536_ has been shown for cells cultured in presence of GM6001 (Fig 3G). However, the concentration of sTM in cell supernatant was remained significantly higher for TM_1-536_ than TM-WT. This data greatly suggests that metalloproteinase action may be one of most determinant parameter involved in TM_1-536_ shedding.

### Shear stress is not a major player for the shedding of TM_1-536_

TM is highly expressed on the capillary vascular endothelium where exist high shear stress forces (2000 à 5000^s-1^). We hypothesized that a short transmembrane domain might be responsible for weaker anchorage of the TM_1-536_ into the cell membrane leading to increased TM shedding when exposed to high shear stress. To evaluate the potential role of shear stress on the release of TM_1-536_ into the circulation, 48h after cell transfection, shear stress (5000s-1) was applied to the COS-1 cells during 0, 2, 5 or 10 min using cone and plate viscometer. Then, the concentration of sTM was determined in supernatant using ELISA method. In the cell supernatant, no significant increase of TM concentration was observed for both TM_1-536_ and TM-WT before and after exposure to shear stress (*p* = 0.229 and 0.078, respectively) (Fig 3H).

### The size of transmembrane domain is determinant for the spontaneous release of TM_1-536_

**Fig 4:**
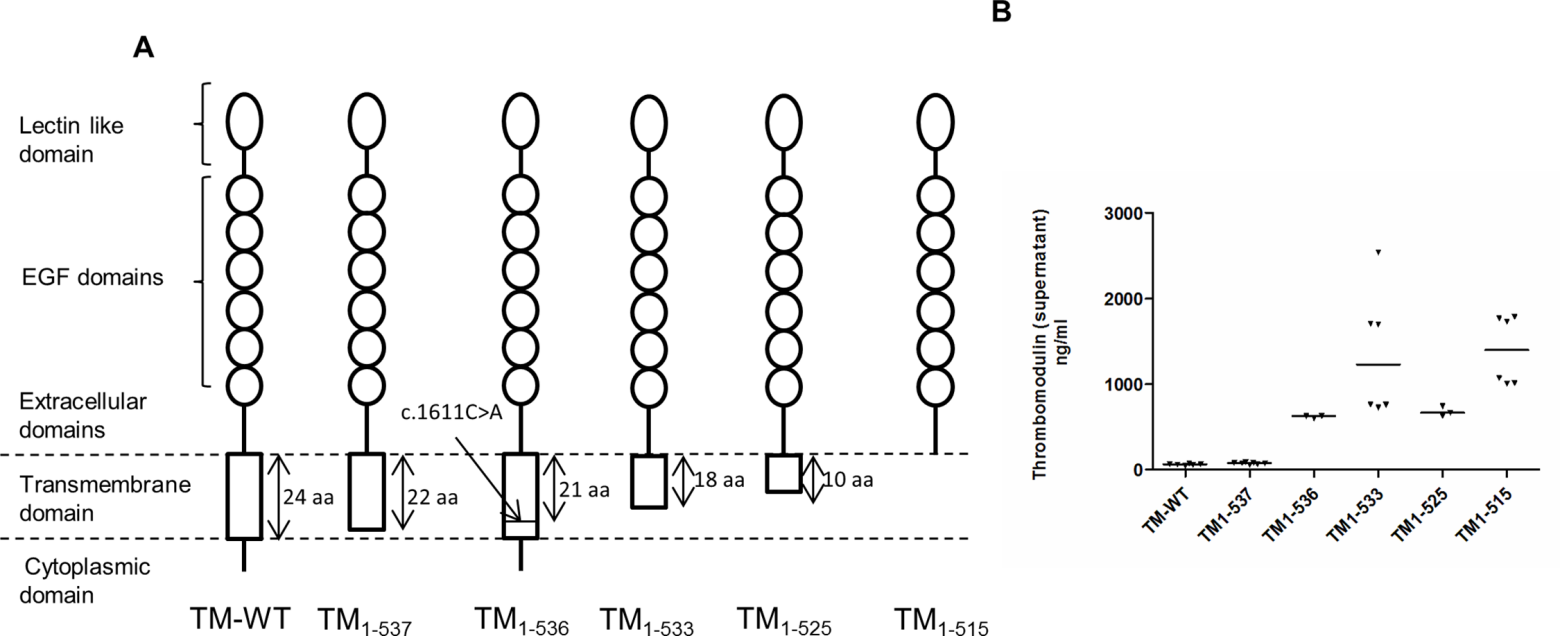
Thrombomodulin shedding according to the size of transmembrane domain. (A) Schematic representation of the different thrombomodulin molecules used in this study. (B) Thrombomodulin shedding according to the size of transmembrane domain. Several thrombomodulin (TM) mutants corresponding to TM with a variable size transmembrane domain ranging from the absence of transmembrane domain (TM_1-515_) to the whole transmembrane domain (TM-WT) were expressed in COS-1 cells. Forty-eight hours after transfection, TM antigen levels were measured by ELISA.

In order to investigate the relationship between the lenght of the transmembrane domain and the shedding of TM, we expressed in COS-1 cells five TM mutants exhibiting variable sizes of their transmembrane domain, ranging from the absence of transmembrane domain (TM_1-515_) to the whole transmembrane domain (TM-WT) (Fig 4A). Forty eight hours after transfection, the concentration of sTM was measured in the cell media (Fig 4B). Higher levels of TM were observed with TM_1-536_, TM_1-539_, TM_1-525_, TM_1-515_ (618 +/- 8, 1366 +/- 303, 678 +/- 33 and 1395 +/- 164 ng/mL, respectively) compared to the TM-WT (62 +/- 4 ng/mL, *p*<0.05). No significant difference was found between TM_1-537_ and TM-WT (62 +/- 4 vs 75 +/- 4 ng/mL, respectively, *p* = 0.0612).

These data showed that the length of the transmembrane domain is a major determinant of TM anchorage into the cells. A truncated transmembrane domain leads to an abnormal and weak anchorage of the protein which may spontaneously be released into the extracellular media independently of the shear stress or the effect of natural proteases.

## Discussion

Most of the mutations in the *THBD* gene were described in patients with thrombosis or atypical hemolytic-uremic syndrome [18–20]. Recently, two independent groups have described a *THBD* c.1611C>A nonsense mutation (p.Cys537Stop) in two families with an autosomal dominant bleeding disorder [12,13]. This mutation results in very high levels of soluble TM in patient’s plasma. We have previously demonstrated that extremely high plasma TM levels enhance fast APC generation and down-regulate FVa and VIIIa and thrombin generation [12,13]. In this study, we investigated the underlying mechanisms responsible for excessive TM shedding from endothelial cells of patients with *THBD* c.1611C>A nonsense mutation.

Individuals with *THBD* c.1611C>A (p.Cys537Stop) mutation have a truncated TM molecule lacking the last three amino acids of the transmembrane domain and the C-terminal intracellular domain [12]. This truncated protein might be retained or degraded into the endothelial cells, or may be highly secreted because of the missing part of the transmembrane domain. We have investigated these hypotheses by performing transient transfections in COS-1 cells followed by immunoblotting and ELISA measurements in cell lysates and supernatants. Western blotting and ELISA performed in the cell lysates confirmed that both TM-WT and TM_1-536_ constructs were equally expressed in COS-1 cells after transfection. Moreover, immunofluorescence analysis showed that both TM-WT and TM_1-536_ were anchored in the cell membrane and expressed at the surface of the cells. However, significantly higher levels of soluble TM were observed in the TM_1-536_ cell media suggesting that the TM_1-536_ was partly secreted in the extracellular medium. These *in vitro* findings are supported by the very high levels of TM measured from the plasma of the patient carrier of this mutation and by the decrease of the membrane-bound TM observed in the index patient’s ovarian tissue [12]. In the present study, ETP was 54% decreased when TM_1-536_ cell supernatant was added into the normal control plasma, demonstrating that soluble TM_1-536_ exhibits anticoagulant activity. This result is in accordance with the bleeding phenotype of the patients exhibiting this mutation and it is in accordance with the structure-function relationship of the molecule, as anticoagulant activity of TM is carried by extracellular EGF domains 4, 5 and 6 [21] which are normal in TM_1-536._

In addition to its anticoagulant activity mediated by APC generation, the TM/thrombin complex exhibits also antifibrinolytic activity due to TAFI activation [22]. Activation of TAFI by the TM/thrombin complex requires EGF domains 3, 4, 5 and 6 which are normal in TM_1-536_ [23]_._ Although the sTM in the serum of patients carrier the c.1611C>A mutation exhibited a complete antifibrinolytic activity [24], the very low levels of thrombin generation observed in propositus might limit the capacity of TM/thrombin complex to activate TAFI and this activity might be probably not sufficient to counteract the anticoagulant activity of sTM.

In physiological conditions, sTM released from cell surface is detectable in plasma at very low levels [6]. Ectodomain shedding is a mechanism used by cells to selectively regulate the expression of surface molecules [25]. Sheddases involved in TM release are poorly understood but *in vitro* data indicated that several proteinases belonging to the MMPs and ADAMs families are involved in TM shedding [9]. We hypothesized that the very high levels of sTM observed in the patients with the c.1611C>A mutation might be explained by a higher vulnerability of the molecule to enzymatic cleavage by proteases. We showed that TM_1-536_ released from COS-1 cells was greatly decreased in the presence of GM6001, a broad-spectrum metalloproteinase inhibitor. This data emphasize the role of these proteases in TM_1-536_ cleavage. We can hypothesize that the missing intracellular and transmembrane domains may lead to a higher vulnerability of TM_1-536_ to the effect of the metalloproteinases compared to TM-WT. Recently, it has been showed that TM is a specific substrate of transmembrane serine protease known as rhomboid-like-2 (RHBDL2; [26,27]). However, the cleavage of TM_1-536_ by the RHBDL2 protease was not explored because the intracellular domain of TM is mandatory for the recognition of the molecule by RHBDL2 and this particular domain is lacking in TM_1-536_ [26]_._ Finally, several inflammatory process are associated with moderate, but statistically significant, increase of TM levels in plasma [12,13] due to the proteolysis of TM by different leukocytes-derived proteases (elastase and cathepsin) and redox-mechanisms [9,28]. Our experiments showed no significant difference of TM shedding between the TM_1-536_ and TM-WT, after exposure of the cells to cathepsin G, elastase and several reducing agents.

We also explored the potential role of shear stress in shedding of this abnormal protein. TM is highly expressed on the capillary vascular endothelium, where exist high shear stress forces (2000 à 5000^s-1^) [29]. The introduction of a negatively charged C-terminus into the hydrophobic lipid bilayer may interfere with stable anchorage of TM to the cell membrane. Our results demonstrated that TM_1-536_ was spontaneously secreted into the cell medium before exposure of the cells to shear stress. After exposure, no significant difference was observed between TM_1-536_ and TM-WT_._

In this study, we explored the relationship between the size of the transmembrane domain and the shedding of TM. A high levels of sTM in cell media was observed for all mutants with a transmembrane domain shortened by at least three amino acids. These results showed that TM shedding was dependent on the presence or absence of an entire transmembrane domain. During its biosynthesis into the endothelial cells, the presence of a truncated transmembrane domain might lead to a certain cell localization defect, therefore a part of the TM might be spontaneously secreted, and not to be properly anchored on the cell membrane.

The main limitation of our work is the use of COS-1 cells which are not constitutively expressing TM. These preliminary data need to be confirmed using an endothelial cell line such as HUVECs.

In conclusion, in this study the pathogenesis of the gain-of-function *THBD* c.1611C>A (p.Cys573X) nonsense mutation was studied. Our results show that the mechanism responsible for TM_1-536_ shedding is complex and involves at last two processes i.e a higher sensitivity to proteolysis induced by metalloproteases and a defect of biosynthesis due to the abnormal transmembrane domain.

## References

[1] Weiler H, Isermann BH. Thrombomodulin. J Thromb Haemost JTH. 2003; 1: 1515–2524. 12871287 10.1046/j.1538-7836.2003.00306.x

[2] Wen DZ, Dittman WA, Ye RD, Deaven LL, Majerus PW, Sadler JE. Human thrombomodulin: complete cDNA sequence and chromosome localization of the gene. Biochemistry (Mosc). 1987; 26: 4350–4357. 10.1021/bi00388a025 2822087

[3] Suzuki K, Kusumoto H, Deyashiki Y, Nishioka J, Maruyama I, Zushi M, et al Structure and expression of human thrombomodulin, a thrombin receptor on endothelium acting as a cofactor for protein C activation. EMBO J. 1987; 6: 1891–1897. 2820710 10.1002/j.1460-2075.1987.tb02448.x PMC553573

[4] Esmon CT, Owen WG. Identification of an endothelial cell cofactor for thrombin-catalyzed activation of protein C. Proc Natl Acad Sci U S A. 1981; 78: 2249–2252. 7017729 10.1073/pnas.78.4.2249 PMC319322

[5] Adams TE, Huntington JA. Thrombin-cofactor interactions: structural insights into regulatory mechanisms. Arterioscler Thromb Vasc Biol. 2006; 26: 1738–1745. doi: 10.1161/01.ATV.0000228844.65168.d1 16728654 10.1161/01.ATV.0000228844.65168.d1

[6] Ohlin A-K, Larsson K, Hansson M. Soluble thrombomodulin activity and soluble thrombomodulin antigen in plasma. J Thromb Haemost. 2005; 3: 976–982. doi: 10.1111/j.1538-7836.2005.01267.x 15869594 10.1111/j.1538-7836.2005.01267.x

[7] Boehme MWJ, Galle P, Stremmel W. Kinetics of thrombomodulin release and endothelial cell injury by neutrophil-derived proteases and oxygen radicals. Immunology. 2002; 107: 340–349. doi: 10.1046/j.1365-2567.2002.01469.x 12423310 10.1046/j.1365-2567.2002.01469.x PMC1782804

[8] Lohi O, Urban S, Freeman M. Diverse substrate recognition mechanisms for rhomboids; thrombomodulin is cleaved by Mammalian rhomboids. Curr Biol CB. 2004; 14: 236–241. doi: 10.1016/j.cub.2004.01.025 14761657 10.1016/j.cub.2004.01.025

[9] Menschikowski M, Hagelgans A, Eisenhofer G, Tiebel O, Siegert G. Reducing agents induce thrombomodulin shedding in human endothelial cells. Thromb Res. 2010; 126: e88–e93. doi: 10.1016/j.thromres.2010.05.006 20605193 10.1016/j.thromres.2010.05.006

[10] Kurosawa S, Stearns-Kurosawa DJ, Kinasewitz GT. Soluble thrombomodulin: a sign of bad times. Crit Care Med. 2008; 36: 985–987. doi: 10.1097/CCM.0B013E318165FDA7 18431290 10.1097/CCM.0B013E318165FDA7

[11] Dharmasaroja P, Dharmasaroja PA, Sobhon P. Increased plasma soluble thrombomodulin levels in cardioembolic stroke. Clin Appl Thromb Off J Int Acad Clin Appl Thromb. 2012; 18: 289–293. 10.1177/1076029611432744 22275395

[12] Dargaud Y, Scoazec JY, Wielders SJH, Trzeciak C, Hackeng TM, Négrier C, et al Characterization of an autosomal dominant bleeding disorder caused by a thrombomodulin mutation. Blood. 2015; 125: 1497–1501. doi: 10.1182/blood-2014-10-604553 25564403 10.1182/blood-2014-10-604553 PMC4342361

[13] Langdown J, Luddington RJ, Huntington JA, Baglin TP. A hereditary bleeding disorder resulting from a premature stop codon in thrombomodulin (p.Cys537Stop). Blood. 2014; 124: 1951–1956. doi: 10.1182/blood-2014-02-557538 25049278 10.1182/blood-2014-02-557538 PMC4168350

[14] Hemker HC, Giesen P, AlDieri R, Regnault V, de Smed E, Wagenvoord R, et al The calibrated automated thrombogram (CAT): a universal routine test for hyper- and hypocoagulability. Pathophysiol Haemost Thromb. 2002; 32: 249–253. doi: 10.1159/000073575 13679651 10.1159/000073575

[15] Dargaud Y, Lienhart A, Negrier C. Prospective assessment of thrombin generation test for dose monitoring of bypassing therapy in hemophilia patients with inhibitors undergoing elective surgery. Blood. 2010; 116: 5734–5737. doi: 10.1182/blood-2010-06-291906 20810929 10.1182/blood-2010-06-291906

[16] Pike GN, Cumming AM, Hay CRM, Bolton-Maggs PHB, Burthem J. Sample conditions determine the ability of thrombin generation parameters to identify bleeding phenotype in FXI deficiency. Blood. 2015; 126: 397–405. doi: 10.1182/blood-2014-12-616565 25911238 10.1182/blood-2014-12-616565

[17] Trossaert M, Lienhart A, Nougier C, Fretigny M, Sigaud M, Meunier S, et al Diagnosis and management challenges in patients with mild haemophilia A and discrepant FVIII measurements. Haemoph. 2014; 20: 550–558. 10.1111/hae.12381 24517184

[18] Delvaeye M, Noris M, De Vriese A, Esmon CT, Esmon NL, Ferrell G, et al Thrombomodulin mutations in atypical hemolytic-uremic syndrome. N Engl J Med. 2009; 361: 345–357. doi: 10.1056/NEJMoa0810739 19625716 10.1056/NEJMoa0810739 PMC3530919

[19] Kunz G, Ohlin A-K, Adami A, Zöller B, Svensson P, Lane DA. Naturally occurring mutations in the thrombomodulin gene leading to impaired expression and function. Blood. 2002; 99: 3646–3653. 11986219 10.1182/blood.v99.10.3646

[20] Kunz G, Ireland HA, Stubbs PJ, Kahan M, Coulton GC, Lane DA. Identification and characterization of a thrombomodulin gene mutation coding for an elongated protein with reduced expression in a kindred with myocardial infarction. Blood. 2000; 95: 569–576. 10627464

[21] Stearns DJ, Kurosawa S, Esmon CT. Microthrombomodulin. Residues 310–486 from the epidermal growth factor precursor homology domain of thrombomodulin will accelerate protein C activation. J Biol Chem. 1989; 264: 3352–3356. 2536746

[22] Bajzar L, Morser J, Nesheim M. TAFI, or plasma procarboxypeptidase B, couples the coagulation and fibrinolytic cascades through the thrombin-thrombomodulin complex. J Biol Chem. 1996; 271: 16603–16608. 8663147 10.1074/jbc.271.28.16603

[23] Conway EM. Thrombomodulin and its role in inflammation. Semin Immunopathol. 2012; 34: 107–125. doi: 10.1007/s00281-011-0282-8 21805323 10.1007/s00281-011-0282-8

[24] Burley K, Whyte CS, Westbury SK, Walker M, Stirrups KE, Turro E, et al Altered fibrinolysis in autosomal dominant thrombomodulin-associated coagulopathy. Blood. 2016; 128: 1879–1883. doi: 10.1182/blood-2016-05-716092 27436851 10.1182/blood-2016-05-716092 PMC5054699

[25] Hayashida K, Bartlett AH, Chen Y, Park PW. Molecular and cellular mechanisms of ectodomain shedding. Anat Rec Hoboken. 2010; 293: 925–937. doi: 10.1002/ar.20757 20503387 10.1002/ar.20757 PMC4621804

[26] Lohi O, Urban S, Freeman M. Diverse substrate recognition mechanisms for rhomboids; thrombomodulin is cleaved by Mammalian rhomboids. Curr Biol CB. 2004; 14: 236–241. doi: 10.1016/j.cub.2004.01.025 14761657 10.1016/j.cub.2004.01.025

[27] Cheng T-L, Wu Y-T, Lin H-Y, Hsu F-C, Liu S-K, Chang B-I, et al Functions of rhomboid family protease RHBDL2 and thrombomodulin in wound healing. J Invest Dermatol. 2011; 131: 2486–2494. doi: 10.1038/jid.2011.230 21833011 10.1038/jid.2011.230

[28] Boehme MW, Deng Y, Raeth U, Bierhaus A, Ziegler R, Stremmel W, et al Release of thrombomodulin from endothelial cells by concerted action of TNF-alpha and neutrophils: in vivo and in vitro studies. Immunology. 1996; 87: 134–140. 8666425 PMC1383979

[29] Mann KG. Thrombin generation in hemorrhage control and vascular occlusion. Circulation. 2011; 124: 225–235. doi: 10.1161/CIRCULATIONAHA.110.952648 21747067 10.1161/CIRCULATIONAHA.110.952648 PMC3138077

